# 5-PC as a Lipid Probe Molecule and as a Second Phospholipid in Binary Phospholipid Mixtures: Saturation Recovery EPR Studies

**DOI:** 10.3390/ijms252312913

**Published:** 2024-11-30

**Authors:** Witold K. Subczynski, Justyna Widomska

**Affiliations:** 1 Department of Biophysics, Medical College on Wisconsin, Milwaukee, WI 53226, USA; subczyn@mcw.edu; 2 Department of Biophysics, Medical University of Lublin, 20-090 Lublin, Poland

**Keywords:** binary mixtures of phospholipids, phospholipid domains, EPR, spin labeling, spin-lattice relaxation, oxygen transport parameter

## Abstract

Mixtures of two phospholipids (PLs) with different main phase transition temperatures were investigated. Host PLs (HPLs) were represented by DMPC, DPPC, DSPC, and DMPE. The admixed PL was the spin-labeled phosphatidylcholine 5-PC(1-palmitoyl-2-(5-doxylstearoyl)phosphatidylcholine), with a unique opportunity to monitor the properties and the local environments of all admixed PL molecules using saturation recovery EPR methods. Below the HPL phase transition temperatures, 5-PC mixes with HPL to form two distinct pools with different rotational diffusion rates. The fluidity of the local environment in these two pools is very different, being more fluid for molecules with greater rotational diffusion rates. Above the HPL phase transition temperature, 5-PC mixes with HPL uniformly. This is independent of the HPL, observed for 5-PC concentrations from 0.25 mol% up to 20 mol% and for the wide temperature range. Assuminga very low concentration of 5-PC is an ideal probe molecule, we can conclude that small fluid phase domains made of HPL molecules are formed below the phase transition temperature of the HPL bilayers. In binary mixtures of HPLs with 5-PC, below the phase transition of HPL bilayers, fluid phase domains are created within the bulk gel phase of HPL lipids by the admixed second PL, namely 5-PC.

## 1. Introduction

Saturation recovery (SR) electron paramagnetic resonance (EPR) spectroscopy was developed by Hyde in the 1970s as a direct way to measure the electron spin-lattice relaxation time (*T*_1_) of paramagnetic molecules [1,2,3]. The quality of *T*_1_ measurements strongly depends on the instrumentation. The first home-builtSR EPR spectrometer was constructed by Hyde and colleagues at the National Biomedical EPR Center, Milwaukee, WI, in 1980 [4,5,6]. A major advancement was made in 1986 with the introduction of the loop-gap resonator [7,8] to the SR spectrometer [8,9]. This instrument was used intensively with nitroxide spin labels to investigate the organization and dynamics of model and biological membranes [9,10,11,12,13,14,15,16] as well as the diffusion and solubility of oxygen in membranous systems [9,17,18,19,20]. Major problems with the application of SR EPR in membrane studies, especially when molecular oxygen is used as a probe molecule, are its ability to measure very short *T*_1_s and its ability to separate components of SR signals with short and long *T*_1_s. In our first measurements, to avoid instrumental noise, we cut a rather significant portion of the SR signal and instead worked on the tail of the decay signal [10,17,18]. Recent major hardware improvements have allowed us to overcome these problems and discriminate SR signals with very short *T*_1_s from those with long *T*_1_s [21]. These improvements delivered a saturating pulse width as narrow as 10 ns at a 1 W power level to the loop-gap resonator. This level of pump power ensures saturation of the sample with the narrowpump pulse widths needed to detect the faster components present in multiexponential signals. Also, the reduction of the receiver dead time after the pump pulse from 300 ns to 100 ns significantly decreased the cut of portion of the SR signals. In some samples, these new capabilities have allowed us to discriminate the short *T*_1_ component, whereas previous capabilities only allowed us to measure one long *T*_1_ component SR EPR signal. Surprisingly, this short *T*_1_ component was observed (in addition to the long *T*_1_ component) for SR EPR signals of 5-PC (1-palmitoyl-2-(5-doxyl stearoyl) phosphatidylcholine) in DMPC (dimyristoylphosphatidylcholine), DPPC (dipalmitoylphosphatidylcholine), DSPC (distearoylphosphatidylcholine), and DMPE (dimyristoylphosphatidylethanolamine) membranes at temperatures below (but not above) the main phase transition temperature. This result was not expected because, under these conditions, membranes should exist as a homogeneous gel phase membrane, and therefore, the nitroxide phosphatidylcholine analog of the DPPC molecules, 5-PC, should also show a single SR EPR signal. These two-component signals were observed for 5-PC/lipid molar ratios as low as 1/400 and for the wide temperature range below the main phase temperature. In our previous experiments, we missed this component for the reasons described above and because the intensity of this component (measured just after the end of the saturation pulse) was much lower than the intensity of the long *T*_1_ component. Thus, cutting the beginning of the SR EPR signal strongly decreases the relative intensity of the fast-decaying component.

**Figure 1:**
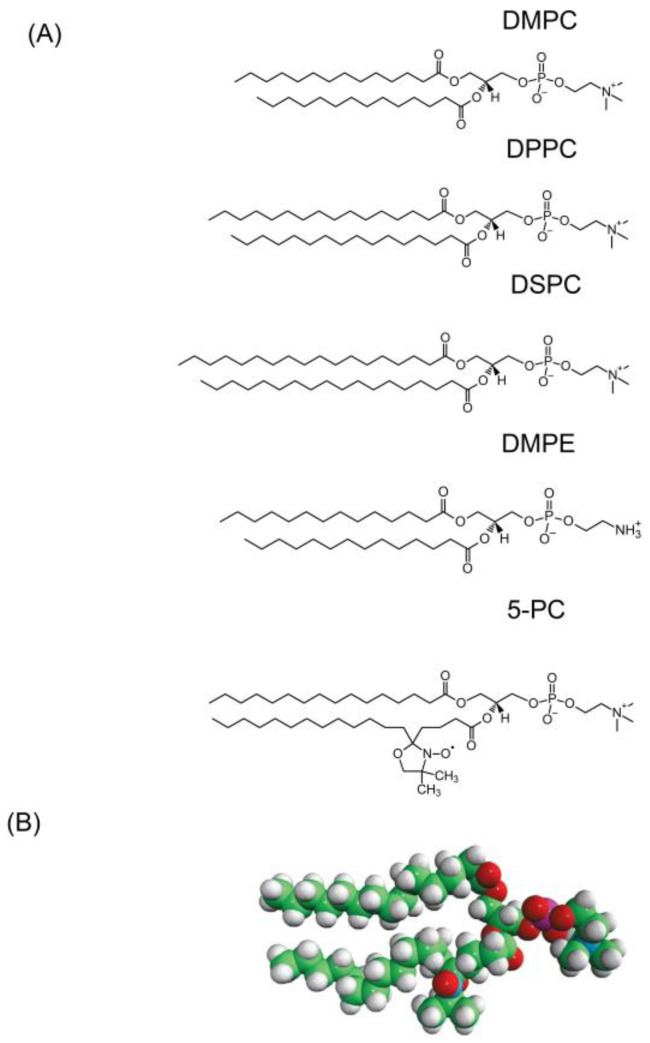
Structures of the HPLs (DMPC (dimyristoylphosphatidylcholine), DPPC (dipalmitoylphosphatidylcholine), DSPC (distearoylphosphatidylcholine), and DMPE(dimyristoylphosphatidylethanolamine)) together with the structure of the admixed PL (5-PC (1-palmitoyl-2-(5-doxyl stearoyl) phosphatidylcholine)) (**A**). Space-filling model of 5-PC (**B**).

To explain these unexpected results, we assumed that 5-PC can be treated not as a probe molecule but as a second component in the binary mixture of two phospholipids (PLs) with different phase transition temperatures. The structure of 5-PC is similar to that of DPPC but with a kink structure (nitroxide label) introduced to the second alkyl chain at the 5C position (see Figure 1). Fortunately, the DSC measurements indicated that the phase transition of the pure 5-PC bilayer occurs between 30.3 °C and 30.7 °C [22]. Thus, for DMPC as a host PL (HPL), 5-PC formed the high-phase transition temperature component. We recognized that because we can use EPR methods to monitor each 5-PC molecule in the membranes, this offers a new, unique experimental opportunity in studies of phase diagrams of binary PL mixtures. Similar opportunities are given by the molecular dynamic (MD) simulation methods. SR EPR methods allow rotational diffusion rates of 5-PC (measured by the 5-PC spin-lattice relaxation rates [*T*_1_^−1^s]) to be obtained. Also, the fluidity of the local environments of 5-PC molecules can be measured through the collision rates of molecular oxygen with the nitroxide moiety of 5-PC. To broaden our understanding of these new results, we extended our experiments to other HPLs (DPPC, DSPC, and DMPE) with phase transition temperatures greater than that for the pure 5-PC bilayer. Thus, for these HPLs, 5-PC will form the low-phase transition temperature component. For all these HPLs, we observed results like those observed for DMPC, namely for temperatures below the phase transition temperatures of the HPL bilayers, 5-PC molecules mixed with HPL molecules to form two distinct pools with different 5-PC properties and with 5-PC molecules with different rotational diffusion rates and fluidities of the local environments. The local environment of the 5-PC molecules was more fluid for molecules with greater rotational diffusion rates. Above the phase transition temperatures of HPLs, 5-PC molecules mixed uniformly with HPL molecules, and only one component SR EPR signal was observed for 5-PC.

Here, we would like to mention that our paper contains two overlapping but somewhat distinct sections. One shows some of the limitations and possible new interpretations of the results obtained by applying lipid spin labels as probe molecules in membrane studies. The second shows new results for the organization of PLs in certain regions of the phase diagrams of mixtures of two PL. In this paper, we present the preliminary data because we think its presentation is both necessary and timely for the above-mentioned reasons. The spirit of this paper is to present empirically obtained data with minimal interpretation.

## 2. Results

### 2.1. Spin Lattice Relaxation Rates of 5-PC in Lipid Bilayers of HPLs

#### 2.1.1. Measurements at the 5-PC/HPL Molar Ratio of 1/400 (at 0.25 mol% 5-PC)

**Figure 2:**
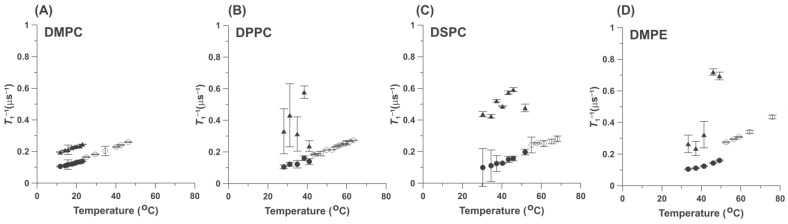
Spin-lattice relaxation rates (*T*_1_^−1^) of 5-PC (0.25 mol%) in HPL bilayers plotted as a function of the temperature. Measurements were performed for (**A**) DMPC, (**B**) DPPC, and (**C**) DSPC bilayers. For (**D**), the DMPE bilayer concentration of 5-PC was 1 mol%. Results obtained below the temperatures of the main phase transitions of the appropriate HPL and assigned to the gel phase membranes (●) and to the fluid phase (▲). Open symbols(o) indicate data obtained abovethe phase transition temperatures of the appropriate HPL andassigned to the fluid phase.

Cumulative results obtained with 0.25 mol% 5-PC for different HPL bilayers (DMPC, DPPC, DSPC) and with 1 mol% 5-PC for DMPE for samples equilibrated with nitrogen gas (0% air) are presented in Figure 2. At temperatures above the HPL phase transition temperatures, one *T*_1_^−1^value was always measured, while at temperatures below the phase transition temperatures, two *T*_1_^−1^s values were obtained. This pattern is observed for the wide temperature ranges, both above and below the HPL phase transition temperatures. Thus, we can conclude that below the HPL bilayer phase transitions, 5-PC (thus the PL admixed to the HPL bilayers) exists in two different pools with different relaxation properties (but see Section 3). Above the HPL bilayer phase transition temperatures, only one pool of 5-PC was observed.

The major conclusions from these results are that 5-PC forms a pool of molecules with a single spin-lattice relaxation rate above the phase transition of the HPL bilayers; thus, 5-PC is dispersed uniformly within the HPL molecules. Because the spin-lattice relaxation rate of the spin labels depends primarily on the rate of rotational motion of the nitroxide moiety [23,24,25], this value describes the dynamics of the lipid molecule to which the nitroxide fragment is rigidly attached. It was also shown that for lipid spin labels, the measured values of the spin-lattice relaxation rates are linear functions of the spin label rotational diffusion rate [26]. Thus, measurements of *T*_1_^−1^ can give qualitative information about the dynamics of lipid molecules to which the nitroxide fragment is attached. Based on this, we can state that above the HPL bilayer phase transition temperatures, all 5-PC molecules possess a single rotational diffusion rate (which is the same for all molecules at the same temperature). Because 5-PC is a probe molecule and an analog of the parent molecule, we can assume that all HPL molecules possess properties similar to those of 5-PC. Below the phase transition, two pools of 5-PC molecules exist, with high and low rotational diffusion rates (Figure 2).

Figure 2 contains data for saturated PC membranes with different acyl chain lengths: 14C for DMPC (Figure 2A), 16C for DPPC (Figure 2B), and 18C for DSPC (Figure 2C). In these measurements, the 5-PC concentration was 0.25 mol%. Figure 2D presents data for saturated polyethylene membranes (DMPE with 14C). The concentration of 5-PC in these measurements was 1 mol%. Results showed that the pattern of the changes of *T*_1_^−1^s of 5-PC is the same regardless of the length of the acyl chains (the thickness of the membrane) and the type of HPL. Single *T*_1_^−1^ values were observed above the phase transition temperature of the membranes made of the HPL, and two *T*_1_^−1^s values were observed below the phase transition temperature. It should be noted that the main phase transition temperatures of the phospholipids (PC) membranes increase with the length of the acyl chains from 23.6 °C for DMPC, 41.2 °C for DPPC, and 55 °C for DSPC [27,28,29,30,31,32]. The phase transition for DMPE occurs at 48 °C [33,34]. The *T*_1_^−1^ describes the property of the 5-PC molecules, namely its rate of rotational diffusion. The single value of *T*_1_^−1^ observed above the phase transition temperatures indicates that at the same temperature, all 5-PC molecules rotate at the same rate. Of course, this rate increases with temperature for all HPL membranes. More complex but interesting patterns of changes of *T*_1_^−1^s were observed below the phase transition temperature of the HPLs. At each temperature, two *T*_1_^−1^ values were observed, indicating that 5-PC properties in these two pools were very different. The rotational diffusion rate of 5-PC molecules in one pool was very low, as is expected for the gel phase membranes, while the 5-PC rotational rate was high in the other pool, comparable to or even greater than that indicated by the *T*_1_^−1^ of 5-PC in the fluid phase (above the phase transition temperature; see Figure 2). When the rotational diffusion rates for the different HPL bilayers are compared at the same temperatures, the values measured for the less fluid components (i.e., those in the gel phase pool) are close. Thus, the fluidity sensed by the 5-PC molecules is independent of the membrane thickness. We cannot make such a comparison for fluid domains created below the phase transition temperatures of the HPL bilayers. Data were unexpectedly scattered, clearly showing the existence of two SR EPR signal components below the phase transition temperatures of the HPLs. That may be due to an increased spin-spin interaction between 5-PCs confined in small but fluid domains. We assume that, in the gel phase bilayers, 5-PC molecules are rather uniformly distributed with concentrations close to 0.25 mol%. Of course, the fluid phase domains cannot be created by only 5-PCs because, in that case, the EPR signals will be broadened to zero amplitude. So, the fluid phase domains contain mixtures of 5-PC and HPL molecules, which possess greater fluidity (rotational diffusion) than the surrounding molecules in the bulk gel phase. The local concentration of 5-PC in these fluid phase domains can be greater than that in the gel phase, and their translational diffusion coefficients can be greater because domains are more fluid (see Section 2.2.2). All these factors should affect (i.e., shorten) the spin-lattice relaxation time of 5-PC.

We would like to explain the unexpected scattering of the data when measurements were performed below the main phase transition temperature of the HPL. It was shown by the measurement of the mobility of 5-doxyl stearic acid spin label (5-SASL) in the gel phase of DPPC membranes that after the temperature drop, the membrane reached equilibrium after a few hours [35]. Here, we did not wait until the samples reached equilibrium, and it can contribute to the scattering of obtained results.

#### 2.1.2. Measurements at 5-PC/HPL Molar Ratios of 1/400, 1/100, 1/9, and 1/4 (at 0.25 mol%,1 mol%, 10 mol%, and 20 mol%)

**Figure 3:**
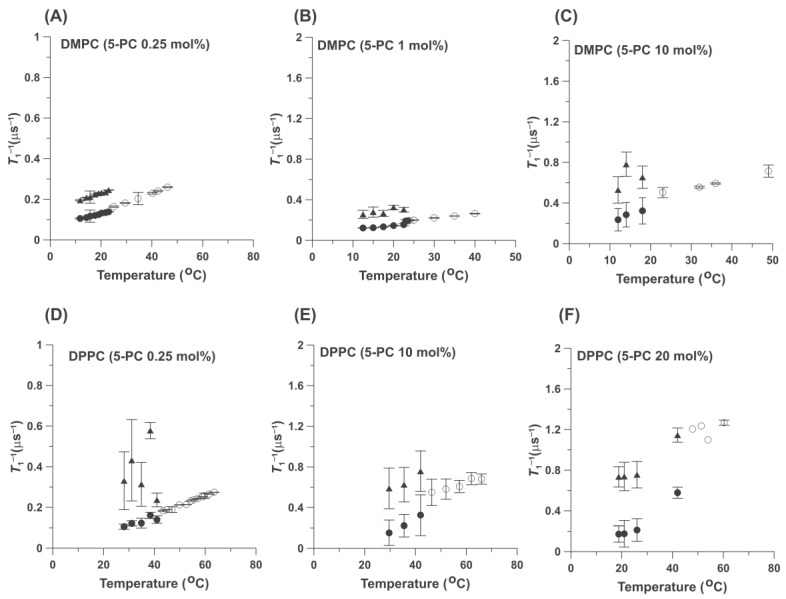
Spin-lattice relaxation rates (*T*_1_^−1^) of 5-PC (0.25 (**A**) 1 (**B**), and 10 mol% (**C**)) in DMPC bilayers and 5-PC (0.25 (**D**), 10 (**E**), and 20 mol% (**F**)) in DPPC bilayers plotted as a function of temperature. Filled symbols indicate data obtained below the phase transition temperatures of the appropriate HPL. Results obtained below the temperatures of the main phase transitions of the appropriate HPL and assigned to the gel phase membranes (●) and to the fluid phase (▲). Open symbols(o) indicate data obtained above the phase transition temperatures of the appropriate HPL and assigned to the fluid phase.

To better understand the effects of admixed 5-PC on the organization and properties of HPL bilayers, we assumed that 5-PC can be treated as a second PL in binary lipid mixtures of PLs. In this approach, two PLs with different main phase transition temperatures were investigated as the HPLs in binary mixtures with admixed 5-PC, which possess a phase transition at 30.5 °C [22]. In these binary mixtures, DMPC formed a low-phase transition temperature component (23.6 °C), and DPPC formed a high-phase transition component (42 °C). To understand the organization of PLs in the investigated binary mixtures, it is important to construct phase diagrams for them that indicate existing phases and domains as a function of temperature and 5-PC content in the HPL bilayers. For these reasons, we performed SR measurements for these two binary mixtures at higher 5-PC contents, up to 20 mol% (up to a molecular ratio of 5-PC/HPL of 1/4). Results presented in Figure 3 indicate that at high 5-PC contents, the pattern of the fluidity changes as a function of temperature in both DMPC and DPPC are qualitatively the same as that observed at 0.25 mol%. Namely, two pools of 5-PC are observed below the phase transition temperatures, while 5-PC indicated that only one homogeneous environment exists above the phase transition temperatures. Quantitative results obtained at 0.25 mol% of 5-PC for magnetically diluted samples (Figure 2) become qualitative because of strong dipole and exchange spin-spin interactions between spin labels at higher concentrations. This is also the reason why we did not use concentrations greater than 20 mol% 5-PC. At higher 5-PC concentrations, measured *T*_1_^−1^ values are significantly greater than those measured for magnetically diluted samples (Figure 2). This can lead to the wrong conclusion about the rotational motion of the spin label and, thus, about the fluidity of their local environment. Fortunately, a different SR approach, namely measurements of the local OTP, allows us to measure the local fluidity around spin labels and is independent of spin label concentration (see Section 2.2 for details). However, the results obtained for the 5-PC concentration region from 0 mol% up to 20 mol% allow us to come to some significant qualitative conclusions that led us to draw the fragmental phase diagrams for the cases when 5-PC formed a binary mixture with HPL at a greater phase transition temperature (with DPPC). These phase diagrams are discussed in Section 2.3.

These properties were independent of the HPLs investigated and were observed for 5-PC concentrations from 0.25 mol% up to 20 mol% and for the wide temperature range above and below the HPL bilayer phase transition temperatures. Two interpretations of the obtained results are discussed. (1) Assuming that 5-PC is an ideal probe molecule at very low concentrations (0.25 mol% and 1 mol%), it can be concluded that small precursor fluid phase domains made of HPL molecules are formed below the phase transition of HPL bilayers. (2) In mixtures of HPLs with the 5-PC that are within the wide temperature range, fluid phase domains are created below the HPL bilayer phase transitions and within the gel bulk HPL lipids by the admixed 5-PC. These results are observed even at a very low concentration of 5-PC (as low as 0.25 mol%) and are in some contradiction with a standard phase diagram of such type of binary PL mixtures.

### 2.2. Local OTP in the HPL Bilayer Measured with 5-PC

#### 2.2.1. Definition and Measurements of the Local OTP

As indicated previously [19,20,23,36], molecular oxygen is the perfect probe molecule for monitoring the properties of the local environment of the nitroxide moiety of the spin label (i.e., 5-PC in these investigations) when measured using the SR EPR technique [19,23]. Therefore, in addition to measuring the spin-lattice relaxation rates, we measured rates of bimolecular collisions between molecular oxygen and the nitroxide fragment of 5-PC expressed as OTP. The oxygen transport parameter (OTP) parameter was introduced (defined) by Kusumi et al. [17] as,

OTP = *T*_1_^−1^(Air, *x*) − *T*_1_^−1^(N_2_, *x*) = A*D*(*x*)*C*(*x*), A = 8π*pr*_0_. (1)

**Figure 4:**
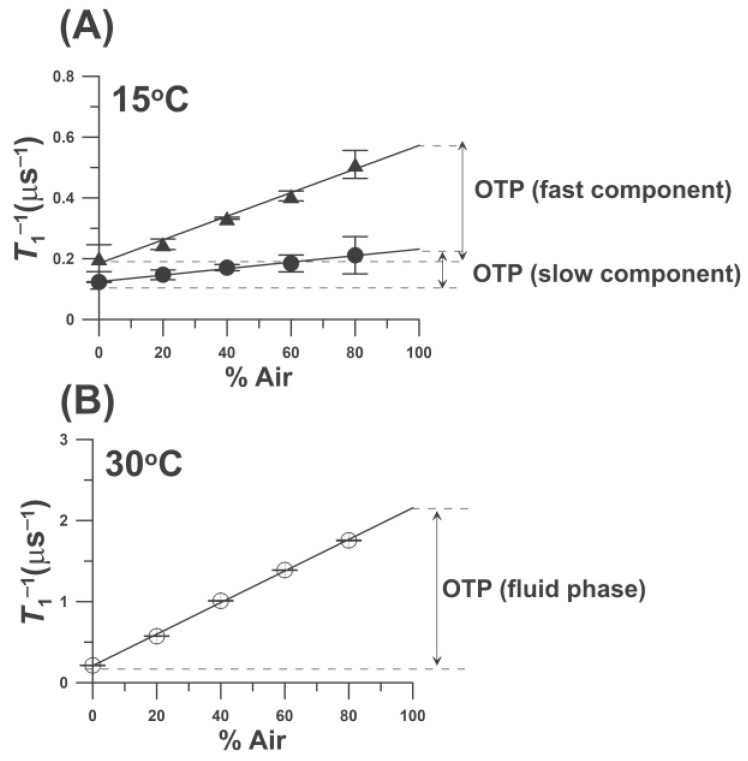
The spin-lattice relaxation rates obtained from fitting the SR EPR signals of 5-PC (1 mol%) in DMPC bilayer (**A**) below the phase transition temperature of DMPC bilayer (at 15 °C), and (**B**) above the phase transition temperature (at 30 °C). In A, signals were successfully fitted to two exponential functions and, in B, to one exponential function. The procedure to obtain OTP by extrapolation to 100% of air is indicated. Results obtained below the temperatures of the main phase transitions of the DMPC and assigned to the gel phase membranes (●) and to the fluid phase (▲). Open symbols(o) indicate data obtained above the phase transition temperatures of the DMPC and assigned to the fluid phase.

*T*_1_^−1^(Air, *x*) and *T*_1_^−1^(N_2_, *x*) are the spin-lattice relaxation rates of the nitroxide in samples equilibrated with atmospheric air and nitrogen, respectively [20,36,37]. OTP is proportional to the product of the local translational diffusion coefficient *D*(*x*) and the local concentration *C*(*x*) of oxygen at the local environment of the nitroxide when the sample is equilibrated with the atmospheric air, *r*_0_ (about 4.5 Å) is the interaction distance between oxygen and the nitroxide [38], and *p* is the probability that an observable event occurs when a collision occurs and is very close to one [17,20,36]. Thus, to obtain the value of the OTP, two SR EPR signals must be measured: one for the deoxygenated sample and the other for the sample equilibrated with air. The value of the OTP is normalized to the atmospheric partial pressure of oxygen in the air surrounding the sample capillary, namely 159.6 mmHg. In the presence of air, *T*_1_s are often too short to be recorded. Thus, to increase the accuracy of the *T*_1_^−1^(Air, *x*) measurements, the value is obtained by extrapolating the linear plot of *T*_1_^−1^ as a function of oxygen concentration (in % air) in the equilibrating gas mixture, and extrapolation results to atmospheric air (100% air). This procedure is shown in Figure 4 for results obtained for one-component (Figure 4B) and two-component SR signals (Figure 4A). Thus, the local environment of the nitroxide moiety is characterized by the OTP parameter, which is very sensitive to the local (around the nitroxide fragment) oxygen diffusion-concentration product. Small variations in the organization of lipids affect oxygen solubility and oxygen diffusion, which can be detected by an increase in the *T*_1_^−1^ of the nitroxide. OTPs were measured for DMPC bilayers with 0.25 mol%, 1 mol% and 10 mol% of 5-PC and for DPPC bilayers with 0.25 mol%, 10 mol%, and 20 mol% 5-PC. Cumulative data and explanations are presented in Section 2.2.2.

#### 2.2.2. Measurements for Different HPLs and at Different 5-PC Contents

**Figure 5:**
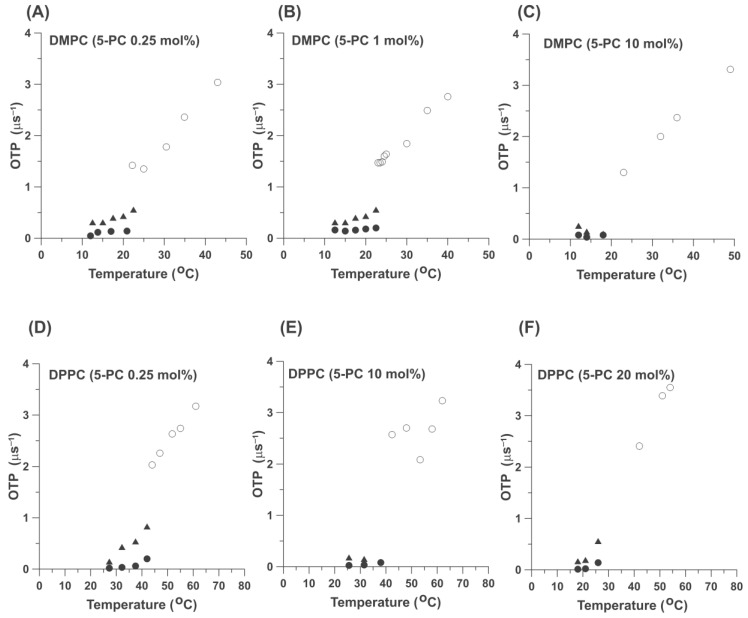
Values of the OTP obtained with 5-PC (0.25 mol% (**A**), 1 mol% (**B**) and 10 mol% (**C**)) in DMPC bilayers and 5-PC (0.25 mol% (**D**), 10 mol% (**E**), and 20 mol% (**F**)) in DPPC bilayers plotted and as a function of temperature. Filled symbols indicate data obtained below the phase transition temperatures of the appropriate HPL. Results obtained below the temperatures of the main phase transitions of the appropriate HPL and assigned to the gel phase membranes (●) and to the fluid phase (▲). Open symbols(o) indicate data obtained above the phase transition temperatures of the appropriate HPL and assigned to the fluid phase.

Measurements were performed to better understand the physical properties of the two pools of 5-PC molecules observed below the HPL phase transition. The OTP provides information about the fluidity of the local environment of 5-PC (that is, the local environment of the nitroxide moiety rigidly attached to the 5-PC molecule) sensed by the diffusion (and solubility) of molecular oxygen. This information is very different than that obtained from measuring the *T*_1_^−1^s of the nitroxide moiety of deoxygenated samples. As shown in Figure 5, the environments around the two pools of 5-PC molecules observed below the HPL phase transitions are also very different. The fluidity of the environment around the 5-PC molecules with greater rotational diffusion rates is greater than that around the 5-PC molecules with lower rotational diffusion rates. Thus, not only do the two pools of 5-PCs exist below the phase transition temperature but these 5-PC molecules are located in two different types of environments (areas) with very different fluidities. The OTP measurements above the phase transition temperature show only a single value, which indicates that the environment around the 5-PC molecules is homogeneous. Additionally, a significant (about twofold) increase in OTP occurs at the phase transition. Further, the *T*_1_^−1^ values of the OTPs increased much more steeply with the increase in temperature. This makes sense because both OTP components—namely, oxygen solubility and oxygen translational diffusion coefficient—increased significantly with temperature. It is also worth noting that the measured OTP values (in contrast with the measured *T*_1_^−1^ values) vary weakly with the 5-PC concentration. This is in line with the definition of OTP (see Section 2.2.1). The high scattering of the OTP values, especially those measured below the phase transition temperatures, results from the difference between the two *T*_1_^−1^ values, which—below the phase transition—can be very small. Changes in OTP with temperature, above and below the phase transition temperature, complement and confirm the conclusions drawn from the measured *T*_1_^−1^ values.

### 2.3. Proposed Phase Diagrams for Binary Mixtures of 5-PC with HPLs

**Figure 6:**
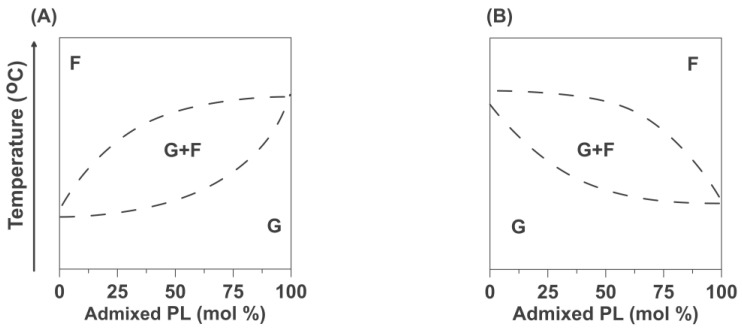
Schematic phase diagrams for the binary PL mixtures for the case when the phase transition temperature of the admixed PL was higher than that of the HPL (**A**) and when the phase transition temperature of the admixed PL was lower than that of the HPL (**B**). F denotes the single fluid phase region, and G denotes the single gel phase region. G + F denotes the region where both phases coexist.

In the investigation of the binary mixtures of PLs, we treated the 5-PC as an admixed PL into the HPL bilayers. The HPLs with phase transition temperatures that were lower and higher than the phase transition temperature of 5-PC were DMPC, DPPC, and DSPC. We confined the discussion to phosphatidylcholines. In Figure 6, we draw typical (schematic) phase diagrams for the mixtures of two phosphatidylcholines with different phase transition temperatures when the admixed PL possess phase transition temperature higher than HPL (Figure 6A, models of mixtures of 5-PC with DMPC) and when the admixed PL possess the phase transition temperature lower than the HPLs (Figure 6B, model of mixtures of 5-PC with DPPC and DSPC). We drown these phase diagrams for binary mixtures of phosphatidylcholines based on the experimental data presented in [32,39,40].

According to the typical phase diagrams for the mixtures of two phosphatydylcholines with different phase transition temperatures, only the single gel phase should exist for temperatures below the main phase transition of DMPC (as in Figure 6A), and only the single fluid phase should exist well above the main phase transition temperature of DPPC and DSPC (as in Figure 6B). Also, the single gel phase should exist for DPPC/5-PC and DSPC/5-PC mixtures well below the phase transition temperatures for these HPLs, especially at low 5-PC concentrations. As expected from schematic phase diagrams (Figure 6), our data confirmed that the single fluid phase exists in the upper regions of the phase diagrams at high temperatures above the main phase transition of HPL. In that phase diagram region, we observe single component SR EPR signals (Figure 2 and Figure 3, single values of *T*_1_^−1^s) and single values of the OTPs (Figure 5). However, for lower temperatures, our results always showed that the gel and the fluid phase coexist. In that phase diagram region, we observe two component SR EPR signals (Figure 2 and Figure 3) and two values of the OTPs (Figure 5). These results were obtained for very low concentrations of 5-PC (0.25 and 1 mol%) as well as for greater 5-PC concentrations, up to 20 mol%.

**Figure 7:**
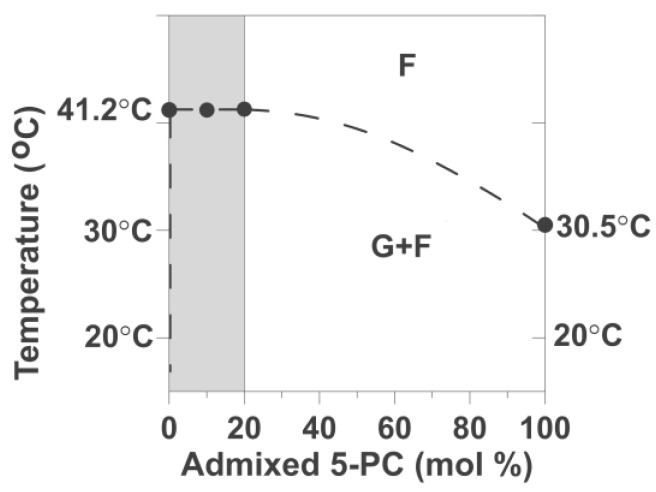
Proposed fragmental phase diagram of 5-PC as it forms a binary mixture with HPL. The phase transition temperature (DPPC) is greater than the phase transition temperature of the pure 5-PC bilayer. Lines separating the region of the homogeneous fluid phase (F) from the region where the gel phase (G) coexists with fluid phase domains (G + F) are indicated. The shadowed area indicates the region for which experimental data were obtained.

Using data for DPPC/5-PC, we constructed the fragmental phase diagram for this binary mixture where existing and coexisting phases were indicated as a function of temperature and 5-PC mixing ratio (Figure 7). Of course, only for the single phases is the molar ratio of 5-PC/HPL equal to the mixing ratio. This was the case for the fluid phase when we recorded data above the phase transition temperature of the DPPC bilayer. Below the phase transition of the DPPC bilayer, 5-PC molecules are distributed between coexisting gel and fluid phases at all investigated conditions, temperatures, and 5-PC mixing ratios. Thus, 5-PC molecules are distributed between these two environments, and their concentration in each environment will be different from the mixing ratio. Typical phase diagrams (Figure 6) for binary mixtures of phosphatidylcholines indicate single-phase regions of the fluid phase and the gel phase separated by the region where the gel phase coexists with the fluid phase. In the phase diagram for the 5-PC/DPPC mixture (Figure 7), we can only indicate the line separating the single-phase region (fluid phase) from the region where the gel phase coexists with the fluid phase. The use of 5-PC as the admixed component in the DPPC bilayer allows us to follow the changes in the organization and properties of the formed bilayer even at a very low concentration of admixed PL (5-PC). At higher concentrations, the spin-spin interactions between 5-PC molecules broadened the EPR spectra and affected (shortened) spin-lattice relaxation times of SR EPR signals. Because of that, we narrowed the region of 5-PC concentration from 0.25 mol% to only 20 mol%. Obtained data allowed the construct of the phase diagram for DPPC/5-PC mixtures only up to 20 mol% of 5-PC (shadowed area in Figure 7), the line separating the fluid phase from coexisting gel and fluid phase is made up based on typical phase diagrams presented in [41,42].

Figure 3 shows the effects of the increased concentration of 5-PC on the measured *T*_1_^−1^ values (and thus on the rotational diffusion coefficient) of 5-PC molecules in DMPC and DPPC bilayers. At all concentrations, the pattern is the same: one-component SR EPR signals above the phase transition temperature of DMPC and DPPC and two-component signals below those temperatures. Most surprising is the fact that two-component SR EPR signals were observed at very low 5-PC/DMPC and 5-PC/DPPC mixing ratios of 1/400 (0.25 mol%) and at temperatures well below the phase transition temperature. Thus, the coexisting gel and fluid phases were created immediately after the addition of the first molecules of 5-PC to the bilayer of the HPL. It allowed us to put the separating line into the phase diagram in Figure 7, just as it overlapped with the temperature axis. In all investigated cases, at all 5-PC concentration regions (from 0.25 up to 20 mol%) and for the wide temperature region (just below and well below the phase transition temperatures), the two components in SR EPR signals of 5-PC were observed, indicating that this spin-label possess two different and well separated rotational diffusion rates, one (with low rotational diffusion rate) characteristic for the gel phase membranes and one with the high diffusion rate. This component we assigned to the fluid phase. As expected, values of *T*_1_^−1^ of 5-PC measured at the same temperature increase significantly with 5-PC concentration. This is a typical shortening of spin-lattice relaxation times occurring with the increased spin label concentration. Major conclusions from this section are confirmed by the measurements of the OTP for DMPC and DPPC bilayers, a parameter that describes the fluidity of the environments of spin labels (Figure 5). This parameter is independent of 5-PC concentration.

## 3. Discussion

If we assume that 5-PC (when used at low concentrations of 0.25 mol% and 1 mol%) is an ideal phospholipid probe molecule that resembles the properties and organization of the HPL molecules in bilayer membranes, we must accept that fluid phase domains are formed in the gel phase of pure HPL bilayers (i.e., below the main phase transition temperature of HPL bilayers). They can possibly form precursors for the fluid phase. These precursor fluid phase domains can be small/unstable with longer lifetimes of the order of *T_1_* (0.1 µs, spin-lattice relaxation time measured in the presence of oxygen)) and/or exchange rates with the bulk surrounding slower than *T*_1_^−1^. They are indicated because of the sensitivity of the SR EPR methods. Above the phase transition temperature, only one homogeneous fluid phase is observed. The fast translational diffusion ensures homogeneity of the fluid phase lipid bilayer. These results were obtained for phosphatidylcholine membranes with saturated acyl chain lengths ranging from 14 to 22 carbon atoms and for polyethylene membranes with saturated acyl chains of 14 carbon atoms. They were confirmed with two types of SR EPR measurements: (1) the spin-lattice relaxation rate (*T*_1_^−1^), which is related to the rotational motion of 5-PC molecules [26] (and thus the rotational motion of HPC molecules), and (2) the oxygen diffusion concentration product (OTP), which indicates the fluidity of the local environment (Figure 2, Figure 3 and Figure 5). Interestingly, the rotational rates of molecules in these fluid phase precursor domains (when compared just below the phase transition temperature of the HPL bilayers) are significantly greater than those in the fluid phase membranes (just above the phase transition temperature); see Figure 3. The rotational diffusion of molecules in the gel phase (i.e., molecules forming the slow rotational diffusion pool) increases when membranes are heated across the phase transition temperature of HPLs, with a very small increase at the phase transition (Figure 2 and Figure 3). Thus, the HPL bilayers melt from the gel phase to the fluid phase bilayers, with a rather small increase in the rotational diffusion of the HPL molecules. Changes in the OTP at the phase transition are much greater than the changes in the rotational diffusion of HPL molecules because they depend on the product of oxygen concentration and oxygen diffusion coefficient, both of which increase at the phase transition. We observed a tenfold increase in the OTP at the phase transition from the gel phase to the fluid phase (Figure 5) and only a twofold increase at the transition from the fluid phase domain (Figure 5). Thus, we know that molecular oxygen as a probe molecule is a very sensitive monitor of bulk membrane fluidity.

In the other interpretation of our results, we should assume that the lipid spin probe, 5-PC, behaves as an independent admixed PL in a binary mixture with HPL molecules. To fit these results to the appropriate phase diagram for binary PL mixtures, we also performed measurements using molecular concentrations that are higher than is typical, i.e., up to 20 mol% of 5-PC. According to our results, independent of the HPL bilayers phase transition temperature and independent of the 5-PC concentration (from 0.25 mol% up to 20 mol%), 5-PC always indicated one homogeneous environment above the phase transition temperatures, and it always indicated two environments below the phase transition temperatures. These results were obtained for the DMPC bilayer, with a phase transition occurring at a temperature (23.6 °C) [28,32,43] that is lower than the temperature (30.5 °C) of the pure 5-PC bilayer [22], and for the DPPC bilayer, with a phase transition occurring at a temperature (42 °C) [28,30,33,44] higher than the temperature (30.5 °C) of the pure 5-PC bilayer. Additionally, these results were repeatedly observed at the wide temperature ranges below and above the phase transition temperatures of both HPLs.

Based on the results, we draw fragmental phase diagrams for 5-PC, forming the binary mixture with HPL with a greater phase transition temperature (with DPPC). In Figure 7, we draw the lines separating regions of the homogeneous fluid phase from the region where the gel phase coexists with the fluid phase for 5-PC concentration up to 20 mol%. This diagram is not compatible with the typical phase diagram presented in Figure 6B, where two lines are drawn to separate the single fluid phase from the coexisting gel and fluid phase and from the single gel phase. In the proposed phase diagrams for the 5-PC/DPPC binary mixture, we cannot draw the second line, which should separate the homogeneous gel phase region from the region where the gel phase coexists with the fluid phase domain. A single gel phase was not detected even well below the phase transition temperature of DPPC and even for extremely low concentrations of 5-PC (as low as 0.25 mol%). Always two environments, the gel phase and the fluid phase, coexisted below the phase transition temperatures of all HPL bilayers.

We would like to note that the presented results were obtained using EPR spin-labeling methods that can be problematic for such types of investigations, namely, at increased 5-PC concentration, the spin-spin interactions between nitroxide moieties affect the observed parameters. Here, the major effect is caused by the Heisenberg spin exchange, which we can observe at greater 5-PC concentrations. The increased spin-lattice relaxation rates observed at 5-PC concentrations of 10 mol% and 20 mol% are not the result of increased rotational diffusion rates of spin-labeled PLs; rather, they are mainly the results of increased collisions between spin labels. This was observed in all phases and domains. However, the OTP did not change with the increased 5-PC concentration. This can be explained by Equation (1) and by the definition of the OTP, which is dependent on the product of the local oxygen concentration and the local oxygen diffusion coefficient around the spin-label. The results presented in Figure 5 provide clear evidence that (from the first approximation) the local environments of 5-PC molecules remain unchanged in all investigated phases and domains when the concentration of 5-PC increases from 0.25 mol% to 20 mol%. Because of the strong effects of spin-spin interactions between 5-PC molecules at high concentrations, we cannot use SR EPR measurements at greater concentrations.

However, the results obtained for the 5-PC concentration region from 0 mol% up to 20 mol% allow us to draw some significant qualitative conclusions. Can the fluid phase domains induced by 5-PC molecules within the bulk gel phase of HPL bilayers always be induced at low concentrations of admixed PLs? Or is this only possible for the special class of admixed PLs? 5-PC is the phosphatidylcholine molecule with the bulky nitroxide moiety attached at the fifth position of one acyl chain [45]. Maybe this group disrupts the ideal mixtures of 5-PCs with rigid, trans conformations molecules of HPLs in gel phase bilayers. This structural nonconformability of neighboring phospholipids supports the segregation of part of 5-PC molecules out from those mixed with the gel phase of HPLs to form fluid phase domains. This nonideal mixing is driven not only by the structural nonconformability of neighboring lipids but also by the thermal energy that regulates the distribution of 5-PC between coexisting domains. At higher temperatures, the relative number of 5-PC molecules in the fluid phase domain increases as compared with that in the gel phase bilayer. Above the phase transition temperature, 5-PC mixes with HPLs to form a homogeneous fluid phase. The structural nonconformability factor is diminished by the segmental motion of the PL molecules. Also, because of the increased lateral diffusion of the molecules, all structural fluctuations (inhomogeneities) can be averaged.

## 4. Materials and Methods

### 4.1. Materials

All PLs and 5-PC (Figure 1) were obtained from Avanti Polar Lipids, Inc. (Alabaster, AL, USA). Other chemicals of at least reagent grade were purchased from Sigma–Aldrich (St. Louis, MO, USA).

### 4.2. Sample Preparation

The membranes used in this work were multilamellar dispersions of the appropriate PL containing 0.25 mol%, 1 mol%, 10 mol%, and 20 mol% of 5-PC and prepared using the film deposition method [46]. A mixture of PL and 5-PC in chloroform was dried with a stream of nitrogen and further dried under a reduced pressure (approx. 0.1 mmHg) for at least 12 h. The 1 ml of buffer (10 mM PIPES [piperazine-N,N’-bis(2-ethanesulfonic acid)] and 150 mM NaCl [pH 7.0]) was added to dried lipid at a temperature above the phase transition temperature of the PL membranes and vortexed vigorously. The lipid dispersion was centrifuged briefly (12,000× *g*, 15 min, 4 °C), and the loose pellet was used for EPR measurement. Structures of host lipids together with 5-PC are shown in Figure 1.

### 4.3. SR EPR

For SR measurements, the sample (the loose membrane pellet) was placed in a capillary (i.d. = 0.6 mm) made of a gas-permeable methylpentene polymer, TPX [19,47]. For measurements of the oxygen transport parameter (OTP), the concentration of oxygen in the sample was controlled by equilibration with the same gas that was used for the temperature control (i.e., a controlled mixture of nitrogen) and dry air-adjusted with flowmeters (Matheson Gas Products model 7631H-604, Matheson, Milwaukee, WI, USA) [17,19,47]. SR signals were obtained at X-b and on a home-built spectrometer with a loop-gap resonator, as described previously [16]. The pulse duration was 0.3 µs. The central-field hyperfine line, which is the most intense, was monitored. Typically, 10^6^ decays were averaged, half of which were off-resonance and differenced for baseline correction, with 2048 data points per decay. Sampling intervals depended on sample, temperature, and oxygen concentration and were either 10 ns or 20 ns. Total accumulation time was about 2–5 min. Recovery curves were fitted by single- and double-exponential functions. The decay times determined from sample to sample were within a precision better than ±3% for single exponentials and ±5% and ±10% for longer and shorter recovery time constants for double exponentials. When necessary, the continuous wave EPR spectra were recorded with a Bruker EMX X-band spectrometer, USA equipped with temperature-control accessories. The results indicate that the SR signals for all recovery curves obtained at temperatures above the HPL phase transitions were satisfactorily fit to a single exponential function. For measurements below the HPL phase transitions, double (but not single) exponential fits were always required. The results of the SR measurements were expressed not as spin-lattice relaxation times (*T*_1_s) but as spin-lattice relaxation rates (*T*_1_^−1^s), which is more convenient for data analysis. Measurements of *T*_1_^−1^s (as described in Section 2.1) were performed systematically for the wide temperature ranges above and below the phase transition temperatures for all the lipid bilayers made of PLs indicated in Figure 1.

## 5. Conclusions

Obtained results allowed us to formulate two basic answers to our questions: (1) If 5-PC is an ideal molecular probe for membrane studies, we must accept the result that, in the gel phase of PL bilayers, a small fluid phase domain can exist within the bulk PL gel phase. (2) In binary mixtures of HPLs, small fluid phase domains in the gel phase are created by the presence of very small amounts of the admixed PL that have higher or lower phase transition temperatures than that of the HPL bilayers. For point (1), as usual, we can state that caution is required when interpreting results obtained using probe molecules. Results connected with point (2) are straightforward because 5-PC actually represents the admixed PL to a bilayer made of HPLs. Of course, the question that remains is whether the investigated system is unique, giving these unique results. Or we can state that results are more uniform and should be observed for broad (different) binary mixtures of PLs. Although we do not have conclusive answers to our questions, we think that their formulation is necessary and timely. We are planning to extend our work to another admixed PL molecule, namely 16-PC, with the pure 16-PC bilayer phase transition occurring between 32 °C and 34 °C. In this molecule, the nitroxide moiety is attached to the 16th carbon atom in the one acyl chain of the PC molecule and is located in the central part of the HPL bilayer. Thus, the problem with the structural nonconformability of the neighboring PLs will be diminished [45].

## Data Availability

Data will be made available on request.
